# Internal Medicine Resident Perceptions of the Barriers to and Facilitators of Optimal Inpatient Care for HIV Prevention of Persons Who Inject Drugs: A Mixed Methods Study

**DOI:** 10.1093/ofid/ofaf124

**Published:** 2025-03-05

**Authors:** Rosemary Bailey, Fauzia Hollnagel, Jessica Tischendorf

**Affiliations:** Division of Infectious Disease, Department of Medicine, University of Wisconsin School of Medicine and Public Health, Madison, Wisconsin, USA; Department of Medicine, University of Wisconsin School of Medicine and Public Health, Madison, Wisconsin, USA; Division of Infectious Disease, Department of Medicine, University of Wisconsin School of Medicine and Public Health, Madison, Wisconsin, USA

**Keywords:** harm reduction, HIV prevention, resident education, substance use disorder, systems engineering

## Abstract

Hospitalizations are an opportunity to offer HIV prevention services to persons who inject drugs. We used mixed methods to describe barriers and facilitators perceived by internal medicine residents to providing these services. Education and electronic medical record interventions can assist our residents in providing this care inpatient.

## BACKGROUND

Persons who inject drugs (PWID) are at risk for infectious complications, including HIV and hepatitis C virus. Harm reduction counseling and access to HIV preexposure prophylaxis (PrEP) and postexposure prophylaxis (PEP) are recommended to prevent infectious sequelae of their substance use disorder [1]. Owing to challenges obtaining regular ambulatory care, hospitalizations should be leveraged to provide these HIV prevention services [2, 3].

Despite the efficacy of PrEP in HIV prevention, use among PWID is low [4]. The barriers to PrEP prescribing among outpatient providers is well described [5–9]; less is known about PrEP prescribing for PWID among inpatient providers [2, 3]. Furthermore, despite their frontline role in caring for these vulnerable patients in our hospitals, little is known about trainee perspectives of harm reduction counseling, PrEP, and PEP for PWID [4, 5, 10]. Addressing barriers to offering these services in the hospital is critical. Failure to equip our residents to provide this care will only perpetuate negative health outcomes in PWID.

We conducted a mixed methods study to characterize internal medicine resident (IMR) perceptions of harm reduction and HIV prevention services among PWID. We sought barriers and facilitators to optimize care that can be used to inform quality improvement work and enhance curricula for residents that aim to optimize HIV prevention among PWID.

## METHODS

### Participants and Setting

We sought participation from internal medicine residents in our mid-sized residency program in a Midwestern academic medical center. All study activities were approved by the University of Wisconsin—Madison Minimal Risk institutional review board. Electronic informed consent was obtained from survey participants and verbal consent from interview participants.

### Survey Design and Distribution

R.B. and J.T. developed the survey questions, which were refined through consultation with the University of Wisconsin-Madison Survey Center. The survey was administered through our school's preferred web-based platform (Qualtrics, Provo, UT). A full copy of the survey is available in Supplementary material. Questions assessed IMRs' perceived importance of, experience with, and comfort with harm reduction counseling and HIV prevention interventions. We also queried their practice in screening for infectious diseases among PWID. Participants were recruited via email and with brief reminders by chief residents during regularly scheduled didactics. Survey responses were incentivized monetarily to promote participation.

### Statistical Analysis

Descriptive statistics were used to summarize the basic features of the survey data. PrEP outcomes were stratified by postgraduate year status using Fisher exact tests.

### Semi-structured Interviews

We conducted 15 semistructured interviews among IMRs. R.B. developed the semistructured interview guide through an iterative process, informed by consultation with the University of Wisconsin—Madison Institute for Clinical and Translational Research qualitative research specialist. We piloted our interview guide with infectious disease fellows and incorporated their feedback into the final guide (available in Supplementary material). Interview participants were recruited electronically by indicating their interest at the completion of the survey. Participants were compensated for their time. Interviews continued until we reached conceptual depth. Interviews were conducted in-person from December 2023 to January 2024 by R.B. Interviews were transcribed verbatim.

Interview transcripts were submitted to consensus coding by 2 researchers (R.B. and J.T.). We applied the Systems Engineering Initiative for Patient Safety (SEIPS) model [11] to characterize IMR interview responses using deductive coding framed by the 5 components of the SEIPS model. The SEIPS model is a framework used to understand the interplay of components of a complex work system, such as an inpatient ward. There are 5 components to the SEIPS model: (1) an *individual* who performs (2) a *task* using (3) *tools or technology* in (4) a *physical environment* within (5) an *organizational* infrastructure.

## RESULTS

### Survey Results

Fifty-nine IMRs (58%) completed the survey. Residents reported a high degree of importance and appropriateness for HIV prevention services, including HIV-PrEP. They lack familiarity with these services and report low comfort and competence in administering and counseling on these services. The majority reported not being familiar at all with the different formulations of HIV PrEP (44.4% for both tenofovir disoproxil fumarate/emtricitabine and tenofovir alafenamide/emtricitabine and 65.5% for cabotegravir) or the Centers for Disease Control and Prevention 2021 Preexposure Prophylaxis for the Prevention of HIV Infection guideline (55.9%). Regarding infectious disease screening, IMRs reported assessing risk of HIV and hepatitis C among PWID at a higher frequency than sexually transmitted infection screening. Many never assessed eligibility for HIV PrEP (22, 38.6%) or HIV PEP (23, 40.4%) regardless of indication (injection use vs sexual risk factors), but more residents feel comfortable prescribing HIV-PrEP for sexual indications than to those who are at risk because of injection drug use. There was no significant difference in these outcomes based on postgraduate year (1, 2, or 3). See Table 1 for full details.

**Table 1.** Internal Medicine Resident Beliefs and Practices in HIV Prevention Interventions and Harm Reduction Counseling for PWID (N = 59, 58% Response Rate)

	Not at all	Slightly	Somewhat	Very	Extremely
How *important* is HIV prevention patient education as part of inpatient care for PWID?	1 (2%)	1 (2%)	6 (10%)	22 (37%)	29 (49%)
How *familiar* are you with harm reduction interventions, such as safer injection counseling, syringe service programs, etc.?	12 (20%)	18 (31%)	23 (39%)	5 (8%)	1 (2%)
How *comfortable* are you with harm reduction interventions, such as safer injection counseling, syringe service programs, etc.?	17 (29%)	16 (27%)	20 (34%)	6 (10%)	0 (0%)
How *important* is HIV-PrEP patient education as part of inpatient care for PWID?	1 (2%)	2 (3%)	7 (12%)	21 (36%)	28 (47%)
How *appropriate* is it to provide PrEP to patients who are eligible at the time of hospital discharge?	0 (0%)	0 (0%)	7 (12%)	23 (39%)	29 (49%)
How *competent* do you feel prescribing HIV PrEP?	14 (23%)	18 (30%)	18 (30%)	10 (17%)	0 (0%)
How *comfortable* do you feel prescribing HIV PrEP to…					
…patients with sexual risk factors for HIV?	4 (7%)	15 (25%)	19 (32%)	16 (27%)	5 (8%)
…patients who inject drugs?	6 (10%)	16 (27%)	22 (37%)	12 (20%)	3 (5%)
	Never	Rarely	Sometimes	Most of the time	Always
In the past 12 mo, when seeing a patient who injects drugs in the inpatient setting, how often have you assessed (through history taking or laboratory screening)…					
…their risk for HIV?	0	10 (18%)	22 (39%)	21 (37%)	4 (7%)
…their risk for hepatitis C?	0	12 (21%)	18 (32%)	23 (40%)	4 (7%)
…their risk for STIs	4 (7%)	8 (14%)	27 (47%)	13 (23%)	5 (9%)
…their eligibility for HIV PrEP?	22 (39%)	21 (37%)	12 (21%)	2 (3.5%)	0
…their eligibility for HIV PEP?	23 (40%)	17 (30%)	15 (26%)	2 (3.5%)	0
In the past 12 mo, when seeing a patient who injects drugs in the outpatient setting, how often have you assessed (through history taking or laboratory screening)…					
…their risk for HIV?	6 (10.7%)	7 (12.5%)	21 (37.5%)	16 (28.6%)	6 (10.7%)
…their risk for hepatitis C?	6 (10.7%)	8 (14.3%)	18 (32.1%)	18 (32.1%)	6 (10.7%)
…their risk for STIs	7 (12.5%)	8 (14.3%)	19 (33.9%)	18 (32.1%)	4 (7.1%)
…their eligibility for HIV PrEP?	16 (28.6%)	19 (33.9%)	14 (25.0%)	6 (10.7%)	1 (1.8%)
In the past 12 mo, when caring for a patient who injects drugs, how often have you …					
…offered HIV PrEP?	33 (60%)	10 (18%)	7 (13%)	4 (7%)	1 (2%)
…offered HIV PEP?	38 (69%)	7 (13%)	7 (13%)	2 (4%)	1 (2%)

Abbreviations: PEP, postexposure prophylaxis; PrEP, preexposure prophylaxis; PWID, persons who inject drugs; STI, sexually transmitted infection.

### Semistructured Interview Results

**Figure 1.**
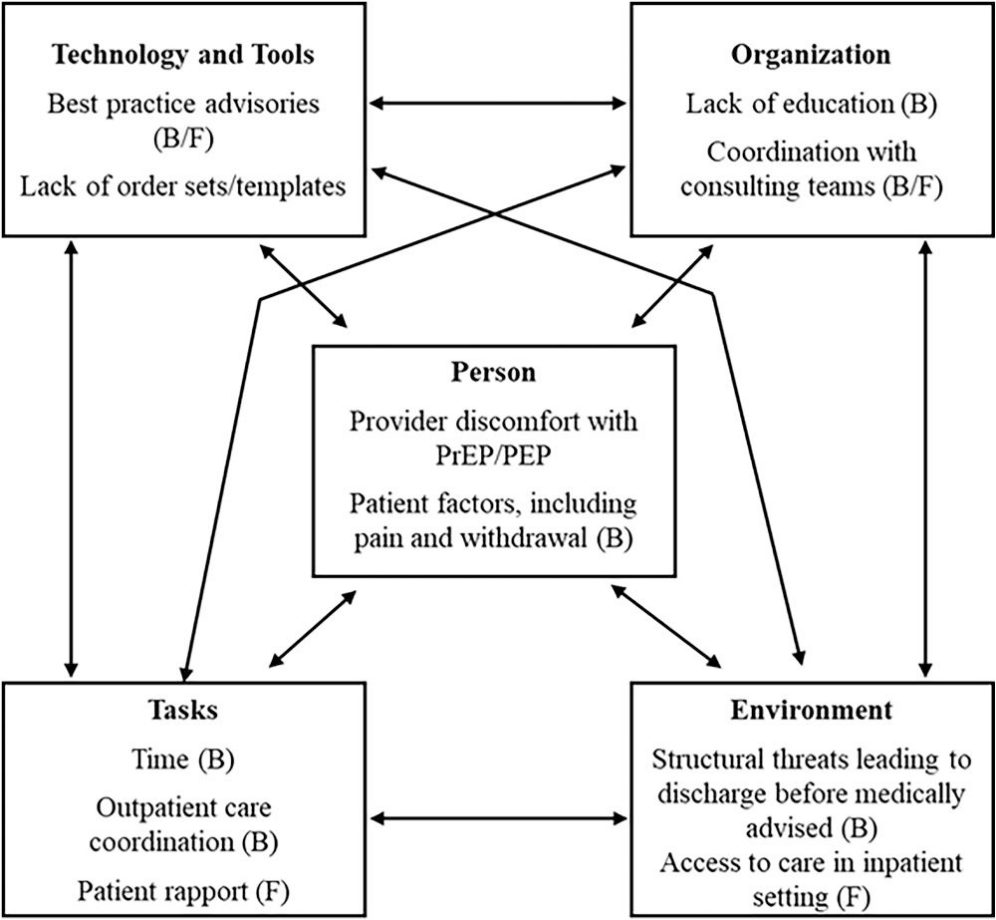
Barriers and facilitators to HIV prevention services, including preexposure prophylaxis (PrEP) and postexposure prophylaxis (PEP), among persons who inject drugs (PWID) as identified by internal medicine residents in our study.

The commonly cited barriers and facilitators to harm reduction counseling and HIV prevention services for PWID admitted to the hospital that emerged from our interviews are summarized in Figure 1. The work system components most referenced as barriers included person (183 references), organization (145), and task (103). The most frequently cited barriers were provider discomfort with prescribing, tension around management of pain and withdrawal symptoms, limited educational resources, and lack of time to address prevention during a hospitalization for acute illness. The work system components most referenced as facilitators included task (125 references), organization (89), and technology and tools (78). The most commonly cited facilitators to optimal prevention practices were rapport with patients, high-quality coordination with consultants, and when they exist, electronic medical record (EMR)–based best practice advisories. Participants identified opportunities for improving care, which include residency curriculum initiatives, EMR alerts for infectious disease screening, and EMR admission and discharge order sets to prompt PrEP/PEP screening, laboratory and medication ordering, and outpatient care coordination. Exemplary quotes are included in the Supplementary material (Supplementary Table 1).

## CONCLUSIONS

IMRs recognize the importance of harm reduction counseling and HIV prevention services, namely HIV-PrEP and PEP, among PWID but lack knowledge, comfort, and competence needed to reliably offer these in the hospital. Several barriers to optimal practice were identified and can be targeted for quality improvement. The most salient barriers were lack of education and inadequate time for HIV prevention counseling, particularly in the setting of higher acuity patient concerns such as pain and withdrawal management. Several means of facilitating greater uptake of these interventions were suggested, including incorporation of these topics into residency curriculum as well as the development of tools within the electronic medical record to serve as a prompt to screen and offer therapy including alerts, admission and discharge order sets, and standardized documentation.

Our findings of inadequate screening and HIV preventive services among PWID admitted to the hospital are not unique [3, 5]. We believe, however, we are the first to describe the perspective of barriers and facilitators of optimal practice in the inpatient setting among physicians in training, a critical frontline workforce in our hospitals across the country. Previous work has identified similar challenges to PrEP prescribing, including lack of education, time constraints, and hesitation to prescribe given unclear outpatient follow-up [6]. In addition, further studies are needed to assess care coordination from inpatient to outpatient to better understand the trend of reversals and abandonments of PrEP, an area that has been explored in the outpatient setting [12]. We are hopeful that educational interventions [5, 10, 13] and systems-level interventions [4, 14, 15] that have proven successful in other settings and with other providers can lead to improved care for PWID admitted to our hospital.

The study has several limitations. First, we conducted this study in a mid-sized Midwestern academic medical center within a geographic region with a relatively low rate of HIV incidence. Like many other places in the country, however, injection drug use remains a public health challenge that will continue to make the risk of HIV acquisition an important consideration for our physicians in training. HIV screening is influenced by the opt-out policy in our state; providers are currently required to obtain and document verbal consent for HIV testing, which may limit generalizability to other locales. As with any study relying on self-report, there is a possibility that residents did not accurately recall their practice patterns. Similarly, author bias may have contributed to how residents responded during the interview process and may have contributed to how responses were subsequently coded. Even considering potential reporting error, the low frequency of infectious disease screening, PrEP and PEP prescribing, and harm reduction counseling reveals a clear area for care optimization. Finally, we did not incorporate the patient perspective. Patient-level barriers are well-described limitations to the uptake of PrEP [2, 9, 16], but we still find it valuable to maximize provider knowledge and comfort to increase the likelihood patients are offered HIV prevention services.

In conclusion, our residents recognize the necessity of HIV prevention services for PWID but require additional support from the educational and health system to operationalize best practices. Our findings can be used to inform quality improvement work for PWID, which must include educational interventions and EMR interventions to facilitate optimal care. By not addressing the barriers to providing HIV prevention services to PWID in the hospital, we are missing a critical opportunity to prevent infectious complications of their substance use.

## Supplementary Material

ofaf124_Supplementary_Data

## Supplementary Data

Supplementary materials are available at *Open Forum Infectious Diseases* online. Consisting of data provided by the authors to benefit the reader, the posted materials are not copyedited and are the sole responsibility of the authors, so questions or comments should be addressed to the corresponding author.
