# Supplementary material for: Internal Medicine Resident Perceptions of the Barriers to and Facilitators of Optimal Inpatient Care for HIV Prevention of Persons Who Inject Drugs: A Mixed Methods Study
Source: Open Forum Infect Dis. 2025 Mar 5;12(4):ofaf124. doi: 10.1093/ofid/ofaf124 (PMC12001333; doi:10.1093/ofid/ofaf124)
Supplement: ofaf124_Supplementary_Data [file ofaf124_supplementary_data.zip › Supplemental Material_IMR survey.pdf]

Block 2

University of Wisconsin - Madison
Research Participant Information and Consent Form

Study Title: Improving the care of patients who inject drugs through a trainee informed educational intervention
Principal Investigator: Jessica Tischendorf (Email: jtischen@medicine.wisc.edu)

Description of the research
You are invited to participate in a research study about the care of patients who inject drugs who are admitted to UW Health facilities. You have been asked to participate because you are a resident physician at UW Health in the Department of Medicine.

The purpose of the research is to explore trainee perceptions of HIV prevention and harm reduction counseling for patients who inject drugs admitted to UW Health facilities.

What will my participation involve?
If you decide to participate in this phase of our research, you will be asked to complete a single survey, which will last approximately 20 minutes.

Are there any risks to me?
We will make all efforts to preserve confidentiality. In the unlikely event of a data breach, there is a possibility your identity could be deduced from your survey responses. Elements of the survey may evoke strong emotions or recollection of negative events.

Are there any benefits to me?
We don't expect any direct benefits to you from participation in this study.

How will my confidentiality be protected?
This study is confidential. Neither your name nor any other identifiable information will be published. Study team members alone will have access to the survey data. We will not ask you to identify yourself by name for this survey. Any quotations used from the open-ended survey responses will be de-identified in such a manner that your response will not be traced to you individually.

Whom should I contact if I have questions?
You may ask any questions about the research at any time. If you have questions about the research you should contact the Principal Investigator Jessica Tischendorf at jtischen@medicine.wisc.edu or study team member Rosemary Bailey at rbailey2@uwhealth.org

If you are not satisfied with response of research team, have more questions, or want to talk with someone about your rights as a research participant, you should contact UW Health Patient Relations at 608-263-8009.

If you decide not to participate or to withdraw from the study, you may do so without penalty.

Your completion of the survey indicates that you have read this consent information, had an opportunity to ask any questions about your participation in this research and voluntarily consent to participate.

If you would like to receive a $25 Amazon gift card for your participation, please provide your email address at the end of the survey through the linked secondary survey. Your email address will not be linked to your survey responses.

Default Question Block

Please choose your level of training:

- PGY1
- PGY2
- PGY3

Have you cared for a patient who was admitted to UW or EMH with complications related to injection drug use during your residency?

- Yes
- No

How **competent** do you feel with prescribing HIV-Pre-exposure prophylaxis (PrEP)?

- Not at all competent
- Slightly competent
- Somewhat competent
- Very Competent
- Extremely competent

How **comfortable** do you feel prescribing HIV-PrEP to

	Not at all comfortable	Slightly comfortable	Somewhat comfortable	Very comfortable	Extremely comfortable
anyone who requests it?	○	○	○	○	○
patients with sexual risk factors for HIV acquisition?	○	○	○	○	○

	Not at all comfortable	Slightly comfortable	Somewhat comfortable	Very comfortable	Extremely comfortable
patients who inject drugs?	○	○	○	○	○

HIV PrEP is available in different formulations such as oral tenofovir disoproxil fumarate/emtricitabine (Truvada), oral tenofovir alafenamide/emtricitabine (Descovy) or injectable cabotegravir (Cabenuva).

How **knowledgeable** do you feel about these different formulations of PrEP?

	Not knowledgeable at all	Slightly knowledgeable	Moderately knowledgeable	Very knowledgeable	Extre knowle
Tenofovir disoproxil fumarate/emtricitabine	○	○	○	○	○
Tenofovir alafenamide/emtricitabine	○	○	○	○	○
Cabotegravir	○	○	○	○	○

The CDC updated their Preexposure Prophylaxis for the Prevention of HIV Infection in the United States in 2021. How **familiar** are you with key changes in this update regarding PrEP?

- ○ Not familiar at all
- ○ Slightly familiar
- ○ Moderately familiar
- ○ Very familiar
- ○ Extremely familiar

In the past 12 months, when seeing a patient who injects drugs in the **inpatient** setting, how often have you assessed (through history taking or laboratory screening)...

	Never	Rarely	Sometimes	Most of the Time	Always
Their risk for HIV?	○	○	○	○	○
Their risk for Hepatitis C?	○	○	○	○	○
Their risk for STIs (Gonorrhea, Chlamydia, Syphilis etc)?	○	○	○	○	○
Their eligibility for HIV PrEP?	○	○	○	○	○
Their eligibility for HIV post-exposure prophylaxis (PEP)?	○	○	○	○	○

In the past 12 months, when seeing a patient who injects drugs in the **outpatient** setting, how often have you assessed (through history taking or laboratory screening)...

	Never	Rarely	Sometimes	Most of the Time	Always
Their risk for HIV?	○	○	○	○	○
Their risk for Hepatitis C?	○	○	○	○	○
Their risk for STIs (Gonorrhea, Chlamydia, Syphilis etc)?	○	○	○	○	○
Their eligibility for HIV PrEP?	○	○	○	○	○
Their eligibility for HIV post-exposure prophylaxis (PEP)?	○	○	○	○	○

In the past 12 months, when caring for a patient who injects drugs, how often have you ...

	Never	Rarely	Sometimes	Most of the Time	Always
Offered HIV PrEP?	○	○	○	○	○
Prescribed HIV PrEP?	○	○	○	○	○
Offered HIV post-exposure prophylaxis (PEP)?	○	○	○	○	○
Prescribed HIV post-exposure prophylaxis (PEP)?	○	○	○	○	○

For the following items, please consider your perceptions of patients who inject drugs.

How **important** is HIV prevention patient education as part of inpatient care for patients who inject drugs?

- ○ Not at all important
- ○ Slightly important
- ○ Moderately important
- ○ Very important
- ○ Extremely important

How **important** is HIV PrEP education as part of HIV prevention patient education during an inpatient stay for patients who inject drugs?

- ○ Not at all important
- ○ Slightly important
- ○ Moderately important
- ○ Very important
- ○ Extremely important

How much do you think PrEP use could improve the health status of patients who are identified as candidates?

- None at all
- A little
- Some
- Quite a bit
- A great deal

How **appropriate** is it to provide PrEP to patients who are eligible at the time of hospital discharge?

- Not at all
- Slightly
- Somewhat
- Very
- Extremely

How **appropriate** is it to provide PrEP in your residency primary care/continuity clinic?

- Not at all
- Slightly
- Somewhat
- Very
- Extremely

Block 1

Certain substance preparation and injection practices are known to reduce the risk of injection related infections.

How **familiar** with these practices (such as safer injection practices, syringe service programs, etc) are you?

- Not at all
- Slightly
- Somewhat
- Very
- Extremely

How **comfortable** do you feel counseling on harm reduction (such as safer injection practices, syringe service programs, etc) for those who use injection drugs?

- Not at all comfortable
- Slightly comfortable
- Somewhat comfortable
- Very comfortable
- Extremely comfortable

How **familiar** are you with the services our addiction medicine colleagues can provide for PWID who are admitted to UWH or EMH?

- Not familiar at all
- Slightly familiar
- Somewhat familiar
- Very familiar
- Extremely familiar

How **well-equipped** do you perceive the UWH or EMH inpatient settings are to offer PrEP for PWID?

- Not at all

- Slightly
- Somewhat
- Very
- Extremely

Thank you for your responses. On the next page, there will be a link to a secondary survey that will allow you to claim your incentive. We would also ask you consider participating in an interview study on this topic. Your responses to the secondary survey cannot be linked to your survey responses.

Block 3

Rosemary Bailey, MD, MS and Jessica Tischendorf, MD, MS at UW-Madison are conducting interviews to learn about barriers and facilitators to HIV prevention and harm reduction practices for patients who inject drugs admitted to internal medicine resident services at UW Hospital and East Madison Hospital. Since you've participated our survey, we invite you to participate in this interview study to gain a deeper understanding of your experience.

We would like to interview you to learn more about your experience providing care to this vulnerable group of patients. The interview will last about 60 minutes and will take place in person or via a video platform. You can choose not to answer any of the questions you are asked and can stop the interview at any time. We will keep your answers confidential and will not share personal information about you with anyone outside the research team.

The interview is confidential and findings will be de-identified, and no one will be able to link your answers back to you. The interview will be recorded with a handheld audio recorder or web-based video platform. Only the researchers will have access to these recordings. Someone hired by the researchers will listen to the recording and write down what people said during the interview. The written copy is called a transcription. The transcription will be saved but the recording will be destroyed. No information that could identify you will be included in the transcription, data analysis or presentation of data.

If you decide to participate in the study, you will receive $100 gift card to thank you for your time. Being in this study is voluntary.

Please contact Rosemary Bailey, MD, MS (rbailey2@uwhealth.org) with questions about this study.

If you would like to participate in the interviews, please follow the link for an additional screening survey. The same link should be followed to claim your $25 incentive for survey participation.

Thank you in advance for considering!

Rosemary Bailey, MD, MS
Jessica Tischendorf, MD, MS

Powered by Qualtrics
