# Supplementary material for: Internal Medicine Resident Perceptions of the Barriers to and Facilitators of Optimal Inpatient Care for HIV Prevention of Persons Who Inject Drugs: A Mixed Methods Study
Source: Open Forum Infect Dis. 2025 Mar 5;12(4):ofaf124. doi: 10.1093/ofid/ofaf124 (PMC12001333; doi:10.1093/ofid/ofaf124)
Supplement: ofaf124_Supplementary_Data [file ofaf124_supplementary_data.zip › Supplemental material_Resident interview guide.docx]

**Resident Interview Guide**

**Title of Study:**Improving the care of patients who inject drugs through a trainee informed educational intervention

**Principal Investigator:**Jessica S. Tischendorf, MD, MS, Assistant Professor (CHS), Division of Infectious Disease, Department of Medicine, University of Wisconsin School of Medicine and Public Health

**Co-Investigators:**Rosemary Bailey, MD, MS, Fellow, Division of Infectious Disease, Department of Medicine, University of Wisconsin School of Medicine and Public Health

Thank you for participating in this interview. You were invited to participate in this study as you are an internal medicine resident physician at UW Health. We are holding these interviews to learn more about your perceptions surrounding HIV prevention and harm reduction counseling among patients who inject drugs admitted to UW Hospital or East Madison Hospital. During this interview, we would like to explore your perception of the barriers and facilitators to optimizing infection prevention care for inpatients who inject drugs.

Today’s interview will last no longer than 60 minutes. This is meant to be a discussion and conversation, I will guide the conversation with questions. You may choose not to answer any questions you do not wish to answer and you can terminate the interview at any time. The researchers will keep information that is shared in the group confidential and will not share any personal information about you outside the research team.

An audio recording will be made of the interview for purposes of transcription*.*Only the researchers and a professional transcription service will have access to these recordings. The researchers or transcription service will listen to the recording and write down what people said during the interview. The written copy is called a transcription. The transcription will be saved but the recording will be destroyed once the transcription is complete. No information that could identify you will be included in the transcription.

With your permission to proceed, we will now start recording and begin our interview.

*********************

*Engagement*

We’d like to begin by having you reflect on recent experiences caring for inpatients who inject drugs:

1. Reflect on your experience throughout your residency. What do you recall about caring for PWID?
   - 1. Are there certain patients that stand out? If so, could you tell me about them.
   1. What challenges do you recall?
      1. In facilitating care
      2. In communicating with other healthcare professionals
      3. In communicating with the patient
      4. In communicating with the patients’ support persons
         1. In arranging appropriate follow-up care
   2. How did you overcome these challenges?
   3. What are the positive things you recall about caring for persons who inject drugs?
      1. What do you enjoy about it?

Now that we’ve explored your experience, let’s turn more specifically to your experience providing HIV prevention services and harm reduction counseling for PWID.

1. Share your typical practice screening for infections among PWID when they are admitted to the hospital.
2. What experience have you had with HIV pre-exposure prophylaxis counseling or prescribing with inpatients who inject drugs?
3. What experience have you had with HIV post-exposure prophylaxis counseling or prescribing with inpatients who inject drugs?
4. Share with me your experience providing PWID with harm reduction counseling.

*Exploration*

Let’s dive deeper into your infectious disease screening practices.

1. Share with me what you know about relevant ID risks among PWID.
2. Share your typical practice screening for infectious diseases when a PWID is admitted to UWH/EMH.

Now, let’s explore your experience discussing or providing HIV PrEP.

1. Share with me what you know about the role of PrEP for PWID.
2. Share past experiences counseling PWID on use of PrEP.
3. What differences do you perceive in your practice of prescribing PrEP for patients who inject drugs when compared to patients who have sexual practice-based indications?

Now, let’s explore your experience discussing or providing HIV PEP.

1. Share with me what you know about the role of PEP for PWID.
2. Share past experiences counseling PWID on use of PEP.
3. What differences do you perceive in your practice of prescribing PEP for patients who inject drugs when compared to patients who have sexual practice-based indications?

Now, let’s explore your experience providing PWID with harm reduction counseling.

1. Have you done this in your practice? Share specific examples if so.
2. Share with me your familiarity with harm reduction principles for PWID.
3. Internal data suggest PrEP/PEP offering and harm reduction counseling are happening infrequently for PWID admitted to UWH and EMH.
4. What reflections do you have on this trend?
5. What are some potential explanations for this trend?
6. What barriers to routine ID screening have you experienced or do you anticipate?
   1. How can these barriers be addressed (facilitators)?
7. What barriers to providing PrEP have you experienced or do you anticipate?
   1. How can these barriers be addressed (facilitators)?
8. What barriers to providing PEP have you experienced or do you anticipate?
   1. How can these barriers be addressed (facilitators)?
9. What barriers to routine harm reduction counseling have you experienced or do you anticipate?
   1. How can these barriers be addressed (facilitators)?

*Exit*

1. What ideas do you have for optimize the care of patients who inject drugs while admitted to UWH or EMH?
   1. What would facilitate improved care?
2. What other reflections would you like to share relating to our discussion?
   - Is there anything you were hoping I would ask about?
