# Supplementary material for: Internal Medicine Resident Perceptions of the Barriers to and Facilitators of Optimal Inpatient Care for HIV Prevention of Persons Who Inject Drugs: A Mixed Methods Study
Source: Open Forum Infect Dis. 2025 Mar 5;12(4):ofaf124. doi: 10.1093/ofid/ofaf124 (PMC12001333; doi:10.1093/ofid/ofaf124)
Supplement: ofaf124_Supplementary_Data [file ofaf124_supplementary_data.zip › Supplemental Table 1 11.26.24.docx]

| **Supplemental Table 1**. Selected quotes from semi-structured interviews with 15 internal medicine residents, categorized by dominant SEIPS work system component. | |
| --- | --- |
| **Person** | |
| Barriers | “I think the biggest overall kind of barrier to communication was that sometimes, either due to the nature of their use disorder or the nature of whatever else was bringing them into the hospital, they were not in a cognitive place to really engage with the Addiction Medicine Team at a level that we might have always wanted.”  “I definitely fall under the category of uncomfortable because I don’t know it specific enough to be, you know, I don’t want to say the wrong thing to the wrong person and disrupt a rapport that I might have with them.  And that comes from a lack of knowledge standpoint for me.” |
| Facilitators | “Some PWID have great social supports, and they have family who, or friends that are at hand and will come in and will help as much as possible.”  “I enjoy talking with patients.  I enjoy providing education.  And so especially if I feel like I'm doing that in a way that is maybe repairing or starting that process of repairing what otherwise would be a negative relationship with healthcare providers, that brings meaning to me.” |
| **Organization** | |
| Barriers | “I know PrEP is super important and that can greatly reduce some of the risk of contracting HIV.  I think one thing that I know from personal experience is that there’s still a stigma around discussing it.”  “I think the biggest limitation there has been finding an outpatient provider who could prescribe the medication and provide the kind of structured regular follow-up that has been shown to be beneficial with persons who have opiate use disorder and inject drugs.”  “It’s just not something that [is] in our curriculum…I don’t even know how long PrEP has been out for.  I would think maybe it’s brand new because it was never mentioned in med school.  I went most of intern year not knowing about it.” |
| Facilitators | “One thing that actually helps a lot is just kind of being a fly on the wall when infectious disease is even talking to the patient, hearing the language they would use, and how they approached talking about substance use disorder.”  “…we’ve had a lot of training in residency about bias language, so that has helped.” |
| **Tasks** | |
| Barriers | “And as the inpatient primary team, it can be difficult, you know, to devote the time resources to having those more in-depth conversations if we have a busy service that we’re trying to cover otherwise.”  “I think in facilitating care, one of the things I’ve encountered that’s difficult is patients who go through withdrawal, whichever substance they’re using when they’re in the hospital.  And so not only are you trying to treat whatever they were admitted for, but also, you’re trying to keep in mind that is this someone who’s going to kind of buy into their treatment plan, or they would want to leave the hospital so they can resume using their substance?” |
| Facilitators | “I just try to get the right team members involved for every scenario, so either social work is probably the biggest supporter for these patients, and they can definitely get certain scenarios resolved or even ease into appointments that they otherwise can’t get there.”  “I think having a standard way of asking questions.  One of the examples I'm thinking of is how I ask code status for patients.  I use the exact same wording, even if I think they're going to be in the [ICU] in two minutes or if they come in with the flu.” |
| **Environment** | |
| Barriers | “Being in the hospital, [the patient is] vulnerable, and they don’t want to have these conversations with strangers, maybe.” |
| Facilitators | “Obviously you don’t want these patients getting sick and coming to the hospital, but also there is this opportunity of: ‘Yes, they’re here. And they’ll be here for several days.’  So not only can you try and address this issue, but you can kind of make sure these things are in line too.” |
| **Technology and Tools** | |
| Barriers | “Not having some kind of prompt to remind me, which I know, you don’t need prompts for everything.  We should use our brains, but it’s always helpful to have something like: ‘this patient has been flagged as this, and they’re not on PrEP. Did you ask them about this?’”  “And if we don’t have materials already put together, then I feel like people are less likely to provide comprehensive harm reduction. “ |
| Facilitators | “I like to be efficient, so if someone gets admitted for a scenario related to IV drug use, maybe there could be an admission order where it has a lot of labs, imaging, and overall discussion of when to use PrEP or PEP, so the provider knows that’s an option there.  There can also be a shared dot phrase that has PEP or PrEP discussion and how to use that could be easily obtained by the providers.”  “I think better education in the IM curriculum could certainly improve that.  Other things that could improve it would be more guideline-based recommendations in the EMR.  That's something that is and will continue to be a great source of therapeutic potential for a lot of patients” |
